# A novel mutation in *GJC2* associated with hypomyelinating leukodystrophy type 2 disorder

**DOI:** 10.5808/gi.22008

**Published:** 2022-06-30

**Authors:** Sajad Rafiee Komachali, Mozhgan Sheikholeslami, Mansoor Salehi

**Affiliations:** 1 Department of Biology, University of Sistan and Baluchestan, Zahedan 98167-45845, Iran; 2 Medical Genetics Research Center of Genome, Isfahan University of Medical Sciences, Isfahan 81759-54319, Iran

**Keywords:** connexin 47, *GJC2*, hypomyelinating leukodystrophy type 2, Pelizaeus–Merzbacher-like disease

## Abstract

Hypomyelinating leukodystrophy type 2 (HLD2), is an inherited genetic disease of the central nervous system caused by recessive mutations in the gap junction protein gamma 2 (*GJC2/GJA12*). HLD2 is characterized by nystagmus, developmental delay, motor impairments, ataxia, severe speech problem, and hypomyelination in the brain. The *GJC2* sequence encodes connexin 47 protein (Cx47). Connexins are a group of membrane proteins that oligomerize to construct gap junctions protein. In the present study, a novel missense mutation gene c.760G>A (p.Val254Met) was identified in a patient with HLD2 by performing whole exome sequencing. Following the discovery of the new mutation in the proband, we used Sanger sequencing to analyze his affected sibling and parents. Sanger sequencing verified homozygosity of the mutation in the proband and his affected sibling. The autosomal recessive inheritance pattern was confirmed since Sanger sequencing revealed both healthy parents were heterozygous for the mutation. PolyPhen2, SIFT, PROVEAN, and CADD were used to evaluate the function prediction scores of detected mutations. Cx47 is essential for oligodendrocyte function, including adequate myelination and myelin maintenance in humans. Novel mutation p.Val254Met is located in the second extracellular domain of Cx47, both extracellular loops are highly conserved and probably induce intramolecular disulfide interactions. This novel mutation in the *Cx47* gene causes oligodendrocyte dysfunction and HLD2 disorder.

## Introduction

Hypomyelinating leukodystrophy type 2 (HLD2), is a genetic disorder of white matter, that nystagmus, progressive spasticity, developmental delay, motor impairments, ataxia, and hypomyelination on the brain are common symptoms in this disorder [1]. The clinical manifestation of HLD2 that also known Pelizaeus–Merzbacher-like disease (MIM #608804) is an insufficient amount of myelin deposition which is observable on brain magnetic resonance imaging (MRI). Primary defects in myelin synthesis and stability are the most common causes of the disease, but myelin damage may also be a factor [1,2]. The majority of individuals with severe hypomyelination begin in infancy or early childhood and develop significant neurological impairments; however, symptoms can also appear in adults [1].

The association between gap junction protein gamma 2 (*GJC2*) gene and HLD2 has been previously reported, homozygous or compound heterozygous mutations in the *GJC2* gene cause HLD2 [3]. The *GJC2* gene, earlier known as *GJA12* (MIM #608803), Also named as *Cx47, HLD2, GJA12, SPG44, CX46.6, LMPH1C, LMPHM3*, and *PMLDAR*, is located on the long arm of the chromosome 1 (1q42.13) [4,5]. Connexin 47 (Cx47, GenBank NP 065168.2) is encoded by the *GJC2* gene and is a member of a highly conserved protein family of connexins [4,5]. The connexins are a group of membrane proteins that form connexon in the cell membrane [6]. Two connexons, each containing six connexin proteins, link across the extracellular space to form a gap junction channel. Cell growth, regulation, and development can all be aided by gap junctions. Ions, intracellular metabolites, and messenger molecules (with a molecular weight of less than 1‒2 kDa) can transfer from one cell’s cytoplasm to its opposing neighbors via these channels [6-8].

In the present study, we report a novel missense homozygous *GJC2* c.760G>A mutation (p.Val254Met) in two cases of HLD2 in a consanguineous family, broadening the range of HLD2-causing *GJC2* mutations. Whole exome sequencing (WES) was used on the DNA sample from the proband who is the affected boy, to identify the mutated gene. Sanger sequencing was used to confirm the discovered mutation in the proband and to investigate this mutation in healthy parents and another affected child.

## Methods

Genomic DNA was extracted from the peripheral blood of the patients and their parents, using innuPREP Blood DNA Mini Kit (Analytika, Jena, Germany) according to the manufacturer’s protocol.

**Fig. 1.**
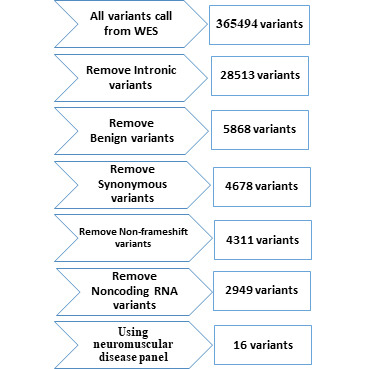
Variant filtering workflow of whole exome sequencing (WES) of patient 1. The number of potential pathogenic Variants was decreased to 16 after filtering by the steps in workflow.

The sample was under WES using Genome Analyzer HiSeq 4000 (101-bp paired-end reads and 100× depth of coverage; Illumina, San Diego, CA, USA) following the manufacturer’s instructions. The library was generated using SureSelect XT Library Prep Kit (Agilent Technologies, Santa Clara, CA, USA). IlluQC tool used for QC of sequencing data. Sequenced reads were aligned to the GRCH37/UCSC hg19 human reference Genome. Also, the post-alignment processing step includes base quality score recalibration (BQSR) was done before variant calling. Then variant calling and VQSR filtering steps were performed, and in the last part of the primary analysis, we annotated the VCF file through ANNOVAR. Exome sequencing identified 365494 annotated variants. To find the disease-causing variation, a filtering FASTQ was set up. Variant filtering is a secondary next-generation sequencing analysis step that includes the stages shown in Fig. 1. Due to eliminating benign variants, only variations with a frequency of less than 1% were chosen. Exome Sequencing Project (ESP), 1000 Genomes (1000G), and Exome Aggregation Consortium (ExAC) data were used to cross-verify the frequencies of discovered variations. We applied a neuromuscular-designed panel to filter the remaining variants. This panel is created by using different databases like CeGat, Fulgent, CENTOGENE, and DisGeNET. We were able to reduce the number of candidate variants to around 16 after applying the filtration steps (Table 1). All 16 variants were checked in OMIM for this study. The variants are then classed as benign, likely benign, a variant of uncertain significance, likely pathogenic, or pathogenic using the American College of Medical Genetics/Association for Molecular Pathology criteria.

Clinical information from a physical examination, laboratory tests or imaging, segregation analysis, genotyping and phenotype correlation, previous publication, or a de novo assessment of the variation were all used to assess the variants. Many of these variants may be ruled out simply by checking the phenotype; in most cases, the reported clinical phenotype of the gene and the patient phenotype are unrelated. It should be emphasized that for autosomal recessive disorders, only homozygous variants were acceptable.

Sanger sequencing was performed by ABI prism 3730 sequencer (Applied Biosystems, Waltham, MA, USA) to validate the pathogenic mutation and segregation the mutation in this family. Mutation Surveyor program version 5.1.2 was used to analyze the sequences (SoftGenetics, State College, PA, USA). Function prediction scores of identified mutation were assessed by PolyPhen2 [9], SIFT [10], PROVEAN [11], and CADD [12].

### Clinical report

The present study involves a Caucasian consanguineous family with two affected children by HLD2 who have been diagnosed with medical evaluations which are standard procedures in individuals with neurological diseases such as MRI. Local ethics committees obtained informed consent from the subjected family.

#### Patient 1

Patient 1 is our proband, and the symptoms of the disease appeared in him with developmental delay. He began occupational therapy at the age of four months and was walking and talking by the age of six. After the age of seven, he began to regress. Since eight years old, he has been unable to walk and can only sit and talk in a few phrases. Nystagmus and vision problems are also issues for him. So far, he hasn't had any seizures.

#### Patient 2

Recurrent seizures, nystagmus, poor vision, and developmental delay were among the signs of the disease, which first appeared when the daughter was three months old. By the age of five with occupational treatment, she was able to sit and pronounce a few words. However, she has regressed and is presently unable to function as a result of recurrent seizures. She has lost her ability to walk, speak, swallow food, and roll. She now experiences seizures every day that are uncontrollable by medication. Seizures become more severe as time passes.

## Results

### Magnetic resonance imaging

**Fig. 2.**
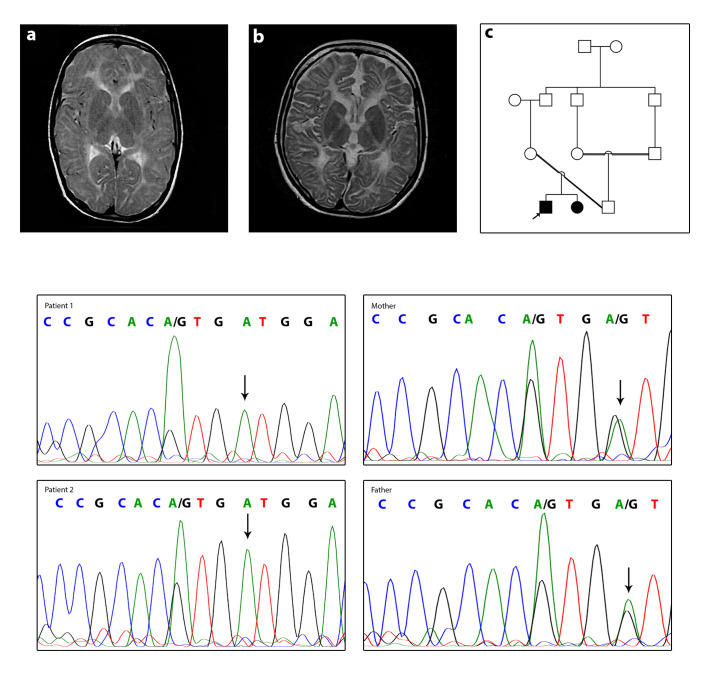
Clinical and molecular features. (A) Axial T2W patient1 (the affected boy). (B) Axial T2W patient2 (the affected girl). Images show hypomyelination around basal ganglia in both patients. (C) Pedigree of the family. (D) The DNA sequences of the patient 1, patient 2, and their parents. Parents are heterozygote for c.760G>A and both patients are homozygote. The mutated allele is shown with arrows.

MRI of the affected boy at the age of 7 years old and the affected girl at the age of 12 years old showed abnormal signal in both cerebral hemispheres white matter high on T2, without mass effect or volume loss mostly due to leukodystrophy. The lateral, 3rd, and 4th ventricles are normal in size and shape midline shift. The brain stem and both cerebellar hemispheres are intact. As well as in the affected girl, a large retention cyst/polyp in the left maxillary sinus is shown in the MRI image (Fig. 2A, B).

### Molecular analyses

Performing WES on proband identified a novel homozygous c.760G>A mutation in the *GJC2* gene. AD—Allele Depth (Read depth for each allele) is 0,116 at c.760G>A mutation, which means read for the reference allele = 0 and the alternative allele = 116. Also, DP—Read Depth is 116 (DP = 116). Sanger sequencing confirmed homozygosity of c.760G>A mutation in the proband and his affected sister and heterozygosity of this mutation in her parents, suggesting it as the putative disease-causing mutation, and autosomal recessive inheritance pattern (Fig. 2C, D).

There is no report of the c.760G>A mutation at the *GJC2* gene in ExAC, 1000G, and other control datasets. We have submitted c.760G>A mutation to ClinVar database, the accession number is SCV001911448. Multiple sequence alignment of the *GJC2* gene by Polyphen2 revealed that Val at position 254 is highly conserved among species. The PolyPhen2 [9], SIFT [10], PROVEAN [11], and CADD [12] results all confirmed that the p.Val254Met mutation is destructive and pathogenic (Table 2).

## Discussion

**Fig. 3.**
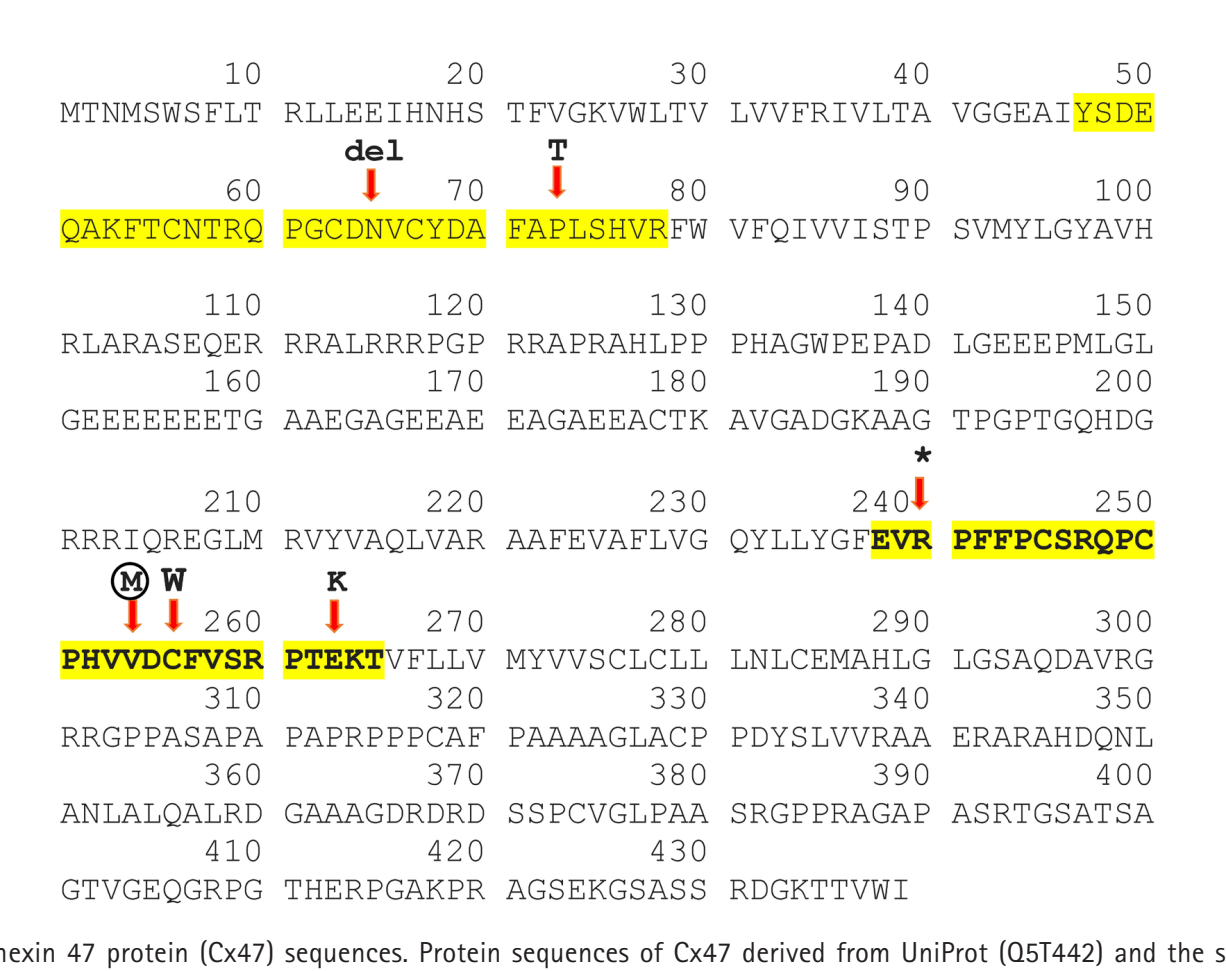
Connexin 47 protein (Cx47) sequences. Protein sequences of Cx47 derived from UniProt (Q5T442) and the sequences of extracellular loops are highlighted with yellow. Based on ClinVar, mutations in both extracellular loops of Cx47 that cause hypomyelinating leukodystrophy type 2 were shown. The novel mutation p.Val254Met described in this study is shown within the circle.

Our patient's novel mutation, p.Val254Met affects a highly conserved position in the second extracellular loop of Cx47 that has four transmembrane domains, two extracellular and three cytoplasmic [6]. It is worth noting that the position of this mutation could change the destiny of protein. It could interrupt the oligomerization of a complete gap junction. Based on ClinVar, other reported missense mutations in the extracellular loops of the Cx47 were depicted in figure 3, which have been associated with HLD2 disease (Fig. 3). Cx47, a significant protein for which crystallography has not yet been performed. Identifying any new mutation in Cx47 can thus be a crucial step toward a better understanding of the protein function.

The remarkable point is that the Cx47 is highly expressed in oligodendrocytes. Oligodendrocytes are a type of glial cells that are responsible for myelination in the central nervous system (CNS) [8,13]. Saltatory conduction is enabled by myelin sheaths, which contribute to the speed-up of action potential conduction in neurons. Also providing energy substrates to neurons is another essential function of oligodendrocytes [14,15].

The heterotypic coupling of Cx30–Cx32 and Cx43–Cx47 forms functional channels between astrocytes and oligodendrocytes [16], and this glial gap junction coupling is crucial for oligodendrocytes function, including adequate myelination and maintenance of myelin in humans [7,17]. Cx47 mutations that cause HLD2 are loss-of-function mutations that probably disrupt or alter the ability to generate functional channels with Cx43, indicating that HLD2 is caused by a loss of oligodendrocytes/astrocytes coupling mediated by Cx47/Cx43 channels [17].

Moreover, in previous studies, the discovery of the altered gap junction protein in the endoplasmic reticulum as a result of I33M mutation in the *Cx47* gene has been suggested as a possible contributor to the pathogenic process of CNS hypomyelination [18]. Also, a transgenic mouse model of Pelizaeus-Merzbacher-like disorder was carrying the human M283T missense mutation in *GJC2*. It has been shown that in oligodendrocytes, expression of the homozygous mutant *Cx47* gene leads to a complicated and diverse neuropathologic phenotype [19]. Last of all, abnormalities in the expression and distribution of the brain connexin like Cx47 play a particular function in the neuropathologies [15].

In Conclusion, in this study, a novel missense mutation, p.Val254Met in the *GJC2* gene, was identified as the cause of HLD2 in two children from a consanguineous family. This gene encodes a gap-junction protein involved in the oligodendrocyte process. This is the first report of p.Val254Met in the *GJC2* gene as responsible for the HLD2. It may be beneficial to investigate this mutation at the protein level. *In vivo* research can also shed light.

## Figures and Tables

**Table 1.** Classification of remaining variants after performing the filtration process

Gene	Position/Variant	Zygosity	Inheritance	Disease	Allele frequency (gnomAD)	Classification	
*HSPG2*	chr1:22183875:G:A	het	AR	Dyssegmental dysplasia, Silverman-Handmaker type	0.000584	VUS by VARSOME and FRANKLIN	
	NM_001291860:exon43:c.C5300T:p.A1767V						
				Schwartz-Jampel syndrome, type 1			
*RYR2*	chr1:237777434:A:G	het	AD	Arrhythmogenic right ventricular dysplasia 2	0.0000161	VUS by VARSOME and FRANKLIN	
	NM_001035:exon37:c.A5006G:p.N1669S						
				Ventricular arrhythmias due to cardiac ryanodine receptor calcium release deficiency syndrome			
				Ventricular tachycardia, catecholaminergic polymorphic, 1			
*MCM10*	chr10:13230915:C:T NM_018518:exon10:c.C1250T:p.A417V	het	AR	Immunodeficiency 80 with or without cardiomyopathy	0.013	Benign by VARSOME and FRANKLIN	
							
*BEST1*	chr11:61722577:AGGCTGG–	het	AD	Microcornea, rod-cone dystrophy, cataract, and posterior staphyloma 2	This variant does not have a gnomAD exomes entry.	Pathogenic by VARSOME and FRANKLIN	
	NM_001363592:exon3:c.153_157del:p.R51Sfs*11			Bestrophinopathy, AR			
							
				Macular dystrophy, vitelliform, 2			
				Retinitis pigmentosa, concentric			
				Retinitis pigmentosa-50			
				Vitreoretinochoroidopathy			
*CAPN1*	chr11:64953448:G:A	het	AR	Spastic paraplegia 76, AR	0.000446	Pathogenic by VARSOME	
	NM_001198868:exon5:c.G517A:p.G173R						
							
						VUS by FRANKLIN	
							
*WNK1*	chr12: 974308:-C	het	AR	Neuropathy, hereditary sensory and autonomic, type II	0.248	Benign by VARSOME and FRANKLIN	
	NM_213655:exon9:c.2173dupC:p.I726Hfs*45		AD	Pseudohypoaldosteronism, type IIC			
*THSD1*	chr13:52951802:T:C	het	AD	Aneurysm, intracranial berry, 12	0.0429	Benign by VARSOME and FRANKLIN	
	NM_199263:exon4:c.A2144G:p.K715R						
*GATM*	chr15:45660340:T:G	het	AR	Cerebral creatine deficiency syndrome 3	0.0000239	VUS by VARSOME and FRANKLIN	
	NM_001482:exon4:c.A603C:p.K201N						
			AD	Fanconi renotubular syndrome 1			
*CTU2*	chr16:88778615:G:A	het	AR	Microcephaly, facial dysmorphism, renal agenesis, and ambiguous genitalia syndrome	0.562	Benign by VARSOME and FRANKLIN	
	NM_001318507:exon6:c.G490A:p.V164M						
*ERCC2*	chr19:45864832:G:A	het	AR	Cerebrooculofacioskeletal syndrome 2	0.0000278	VUS by VARSOME and FRANKLIN	
	NM_001130867:exon11:c.C1115T:p.P372L			Trichothiodystrophy 1, photosensitive			
				Xeroderma pigmentosum, group D			
*NBAS*	chr2:15614274:G:A	het	AR	Infantile liver failure syndrome 2	0.0000239	VUS by VARSOME and FRANKLIN	
	NM_015909:exon15:c.C1516T:p.R506W						
				Short stature, optic nerve atrophy, and Pelger-Huet anomaly			
*PCCB*	chr3:136046527:A:T	het	AR	Propionicacidemia	This variant does not have a gnomAD exomes entry.	Likely Pathogenic by VARSOME	
	NM_000532:exon13:c.A1351T:p.T451S						
						VUS by FRANKLIN	
*AP4M1*	chr7: 99704060:G:T	het	AR	Spastic paraplegia 50, autosomal recessive	This variant does not have a gnomAD exomes entry.	VUS by VARSOME and FRANKLIN	
	NM_001363671:exon14:c.G1081T:p.A361S						
*CLCN1*	chr7:143048771:C:T	het	AD	Myotonia congenita, dominant	0.00288	Benign by VARSOME	
	NM_000083:exon23:c.C2680T:p.R894X						
			AR				
				Myotonia congenita, recessive		Pathogenic by FRANKLIN	
			-	Myotonia levior, recessive			
*RIMS2*	chr8:105105846:C:T	hom	AR	Cone-rod synaptic disorder syndrome, congenital nonprogressive	0.201	Benign by VARSOME and FRANKLIN	
	NM_001348498:exon19:c.C2992T:p.R998C						
*GJC2*	chr1:228346219:G:A	hom	AR	Spastic paraplegia 44, AR	This variant does not have a gnomAD genomes entry.	VUS by VARSOME	
	NM_020435:exon2:c.G760A:p.V254M						
						Likely Pathogenic by FRANKLIN	
			AR	Leukodystrophy, hypomyelinating, 2			
			AD	Lymphatic malformation 3			

het, heterozygous; AR, autosomal recessive; AD, autosomal dominant; hom, hoozygous; VUS, variant of uncertain significance.

**Table 2.** Prediction of p.Val254 Met substitution effect

Prediction tools	Score	Prediction
Polyphen2 HumDiv	1	Probably damaging
Polyphen2 HumVar	1	Probably damaging
Provean	‒2.73	Deleterious
SIFT	0.002	Damaging
CADD-phred score	26.3	Deleterious
